# Solar‐Powered AEM Electrolyzer via PGM‐Free (Oxy)hydroxide Anode with Solar to Hydrogen Conversion Efficiency of 12.44%

**DOI:** 10.1002/advs.202401782

**Published:** 2024-04-24

**Authors:** Jun Seok Ha, Youngtae Park, Jae‐Yeop Jeong, Seung Hun Lee, Sung Jun Lee, In Tae Kim, Seo Hyun Park, Hyunsoo Jin, Soo Min Kim, Suwon Choi, Chiho Kim, Sung Mook Choi, Bong Kyun Kang, Hyuck Mo Lee, Yoo Sei Park

**Affiliations:** ^1^ Department of Advanced Material Engineering Chungbuk National University Chungdae‐ro 1, Seowon‐Gu Cheongju Chungbuk 28644 Republic of Korea; ^2^ Department of Materials Science and Engineering Korea Advanced Institute of Science and Engineering (KAIST) Daejeon 34141 Republic of Korea; ^3^ Hydrogen Research Department Korea Institute of Energy Research (KIER) 152 Gajeong‐ro Yuseong‐gu Daejeon 34129 Republic of Korea; ^4^ Department of Hydrogen Energy Materials Surface & Nano Materials Division Korea Institute of Materials Science (KIMS) Changwon 51508 Republic of Korea; ^5^ Department of Materials Science and Engineering Pusan National University Busan 46241 Republic of Korea; ^6^ Department of Urban, Energy, and Environmental Engineering Chungbuk National University Chungdae‐ro 1 Seowon‐Gu, Cheongju, Chungbuk 28644 Republic of Korea; ^7^ Department of Mechanical & Materials Engineering Worcester Polytechnic Institute 100 Institute Road Worcester MA 01609 USA; ^8^ Nano Electronic Materials and Components Research Center Gumi Electronics and Information Technology Research Institute Sandongmyeon Gumi 39171 Republic of Korea; ^9^ Advanced Materials Engineering University of Science and Technology (UST) Daejeon 34113 Republic of Korea; ^10^ Department of Electronic Materials, Devices, and Equipment Engineering Soonchunhyang University 22, Soonchunhyang‐ro Asan City Chungnam 31538 Republic of Korea; ^11^ Department of Display Materials Engineering Soonchunhyang University 22, Soonchunhyang‐ro Asan City Chungnam 31538 Republic of Korea

**Keywords:** anion exchange membrane water electrolysis, electrocatalysis, hydrogen energy, solar to hydrogen, water splitting

## Abstract

Water electrolyzers powered by renewable energy are emerging as clean and sustainable technology for producing hydrogen without carbon emissions. Specifically, anion exchange membrane (AEM) electrolyzers utilizing non‐platinum group metal (non‐PGM) catalysts have garnered attention as a cost‐effective method for hydrogen production, especially when integrated with solar cells. Nonetheless, the progress of such integrated systems is hindered by inadequate water electrolysis efficiency, primarily caused by poor oxygen evolution reaction (OER) electrodes. To address this issue, a NiFeCo─OOH has developed as an OER electrocatalyst and successfully demonstrated its efficacy in an AEM electrolyzer, which is powered by renewable electricity and integrated with a silicon solar cell.

## Graphical abstract

Incorporating cobalt into NiFe(oxy)hydroxide significantly reduces the electrochemical activation barrier for an oxygen evolution reaction, thereby substantially diminishing activation losses in an AEM electrolyzer and resulting in high efficiency. Furthermore, the integrated system of the AEM electrolyzer and silicon solar cells demonstrates high solar‐to‐hydrogen efficiency.

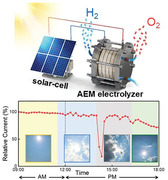

## 1 Introduction

An effective with the ongoing expansion of the renewable energy industry, the supply of electricity generated from renewable sources is projected to experience exponential growth.^[^ ^1^ ^]^ However, this growth has intensified concerns surrounding energy storage.^[^ ^2^ ^]^ Currently, renewable energy is primarily stored in battery energy storage systems (B‐ESS), but their high cost and limited capacity flexibility pose significant challenges. Therefore, there is an urgent need for an energy storage system that is both cost‐effective and capable of accommodating high storage capacity.

Power‐to‐gas (P2G) is a widely recognized technology that facilitates the conversion of electrical energy into chemical fuels.^[^ ^3^ ^]^ Water electrolysis, one of the P2G, enables the conversion of electricity into the chemical energy of hydrogen, allowing renewable energy to be stored as hydrogen fuel.^[^ ^4^ ^]^ This energy storage system using water electrolysis is called a hydrogen energy storage system (H‐ESS) and offers good capacity flexibility. Despite its advantages, the commercialization of H‐ESS has been delayed, and B‐ESS remains the primary choice for storing renewable energy. The major obstacle hindering the use of H‐ESS is the significant power loss in the process of converting electricity into the chemical energy of hydrogen. This challenge can be overcome by developing a high‐performance water electrolysis system.

Alkaline water electrolyzers (AWE) and proton exchange membrane water electrolyzers (PEM electrolyzers) represent two prominent types of water electrolysis systems. Specifically, the PEM electrolyzer employs a zero‐gap configuration, where the electrode distance approaches zero, and utilizes highly active platinum group metals (PGMs) as electrocatalysts. This configuration enables high energy conversion efficiency, making it suitable for integration with H‐ESS.^[^ ^5^ ^]^ However, the use of PGMs leads to high hydrogen production costs, which serves as a drawback. A recently developed technology, the anion exchange membrane water electrolyzer (AEM electrolyzer), combines the advantages of AWE and PEM electrolyzers.^[^ ^6^ ^]^ It achieves high efficiency by employing a zero‐gap configuration and utilizes non‐PGM electrocatalysts for cost‐effective hydrogen production. However, non‐PGM electrocatalysts exhibit insufficient catalytic activity compared to PGM electrocatalysts, and current AEM electrolyzers experience significant voltage losses originating from the oxygen evolution reaction (OER). Therefore, there is a need to develop highly active electrocatalysts for the OER to connect AEMWE and H‐ESS effectively. According to research conducted so far, transition metal‐based layered double hydroxides (LDHs) and (oxy)hydroxides are known as the best electrocatalysts for OER.^[^ ^7^ ^]^ Among them, NiFe(oxy)hydroxide has demonstrated exceptional OER activity. However, to achieve higher energy conversion efficiency in the AEM electrolyzer, further improvements in OER activity are required. One approach to enhance OER activity is by introducing a third element into NiFe(oxy)hydroxide.^[^ ^8^ ^]^ Cobalt is commonly employed due to its ability to modulate the electronic structure of nickel and iron, resulting in improved OER activity.^[^ ^9^ ^]^ Therefore, the use of NiFeCo‐based (oxy)hydroxide could significantly enhance the performance of the AEM electrolyzer.

To utilize the developed electrocatalyst in the AEM electrolyzer, an electrode manufacturing process is necessary. This process usually includes mixing the electrocatalyst and polymer binder (PTFE and ionomer) in the appropriate ratio and then coating the mixture onto a substrate.^[^ ^10^ ^]^ However, optimizing the ratio of the electrocatalyst to polymer binder, as well as controlling various parameters, such as temperature, pressure, and time for coating the catalyst layer, make the electrode manufacturing process more complex and increase its cost.^[^ ^10^, ^11^ ^]^ In particular, commercial AEM electrolyzer stacks require dozens of large‐area electrodes (>64 cm^2^ per electrode),^[^ ^12^ ^]^ indicating that a simple and cost‐effective electrode manufacturing process is crucial. In this work, we developed NiFeCo─OOH as an OER electrocatalyst for an AEM electrolyzer and realized the AEM electrolyzer powered by renewable electricity by constructing a system integrated with the AEM electrolyzer and a silicon solar cell. The NiFeCo─OOH was grown on commercial nickel foam by galvanic corrosion and directly used as an OER electrode (anode). The introduction of Co into NiFe─OOH improved the OER activity by lowering both the onset potential and activation barrier for OER while maintaining the OER kinetics. The AEM electrolyzer equipped with NiFeCo─OOH exhibited a lower activation loss than that of NiFe─OOH and achieved high performance, with ≈2.0 A cm^−2^ at 1.809 V_cell_. Furthermore, we successfully demonstrated a high‐performance AEM electrolyzer powered by renewable electricity generated from commercial solar cells. This integrated system showed a high solar‐to‐hydrogen (STH) efficiency of ≈12.44%.

## 2 Results and Discussion

**Figure 1:**
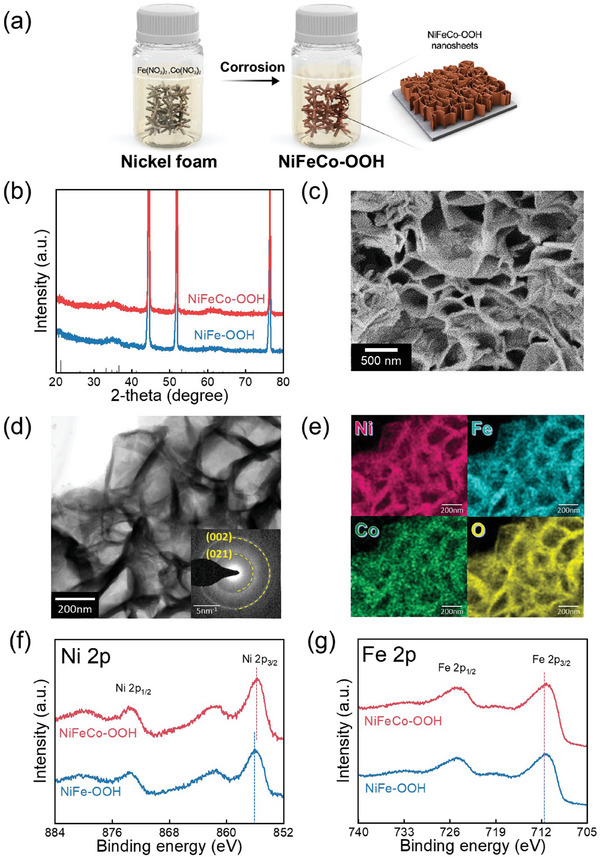
Characterization of NiFeCo─OOH. a) Schematic illustration for the preparation of NiFeCo─OOH. b) XRD patterns of NiFe─OOH and NiFeCo─OOH. c) SEM images of NiFeCo─OOH. d) TEM images of the NiFeCo─OOH with SAED pattern. e) EDS elemental mapping images of the NiFeCo─OOH: Ni(red), Fe(blue), Co(yellow), O(purple). High‐resolution XPS spectrum of f) Ni 2p and g) Fe 2p.

**Figure** **1**a presents the schematic depicting the preparation process of NiFeCo─OOH through galvanic corrosion engineering. Figure S1 (Supporting Information) shows photographs of NiFe─OOH and NiFeCo─OOH. XRD patterns of NiFeCo─OOH and NiFe─OOH were obtained to identify the crystal phase (Figure 1b). The strong diffraction peaks at 44°, 52°, and 76° indicated the metallic Ni (JCPDS No.96‐901‐2972).^[^ ^13^ ^]^ The XRD patterns of NiFe─OOH were indexed as FeOOH (JCPDS No. 01‐081‐0463).^[^ ^14^ ^]^ Interestingly, despite the incorporation of Co to NiFe─OOH, the XRD pattern of NiFeCo─OOH resembled that of NiFe─OOH. FE‐SEM images were acquired to examine the surface morphology of NiFeCo─OOH and NiFe─OOH, as shown in Figure 1c and Figure S3 (Supporting Information). NiFe─OOH exhibited a typical nanosheet shape, and NiFeCo─OOH showed a similar nanosheet morphology to NiFe─OOH.^[^ ^15^ ^]^

TEM images also revealed nanosheet shapes for both NiFe─OOH and NiFeCo─OOH (Figure 1e; Figure S2, Supporting Information). The selected area electron diffraction (SAED) patterns of the NiFeCo─OOH and NiFe─OOH were indexed as (021) and (002) planes of FeOOH. These SAED patterns, along with the XRD results, indicate that the introduction of Co into NiFe─OOH does not impact the crystal structure. The elements distribution of Ni, Fe, Co, and O was observed by TEM‐EDS mapping and showed that all elements were uniformly distributed (Figure 1e; Figure S1b, Supporting Information). Furthermore, EDS analysis confirmed that NiFe─OOH comprised ≈30 at.% (Ni) and ≈70 at.% (Fe), while NiFeCo─OOH consisted of ≈26 at.% (Ni), ≈69 at.% (Fe), and ≈5 at.% (Co). To investigate the influence of Co on vibrational modes, Raman spectra of NiFe─OOH and NiFeCo─OOH were obtained, as shown in Figure S4 (Supporting Information). A prominent peak around 670 cm^−1^, associated with Fe─O vibrations in the FeOOH phase, was observed.^[^ ^16^ ^]^ Additionally, peaks at 470 and 546 cm^−1^, corresponding to Ni─O vibrations in the NiOOH phase, were observed. Incorporating Co into NiFe─OOH, a red shift was observed, suggesting an increase in bond length between Ni/Fe─O and a decrease in bond energy. These findings indicate that introducing Co into NiFe─OOH leads to alterations in the bonding energy between metal and oxygen.

**Figure 2:**
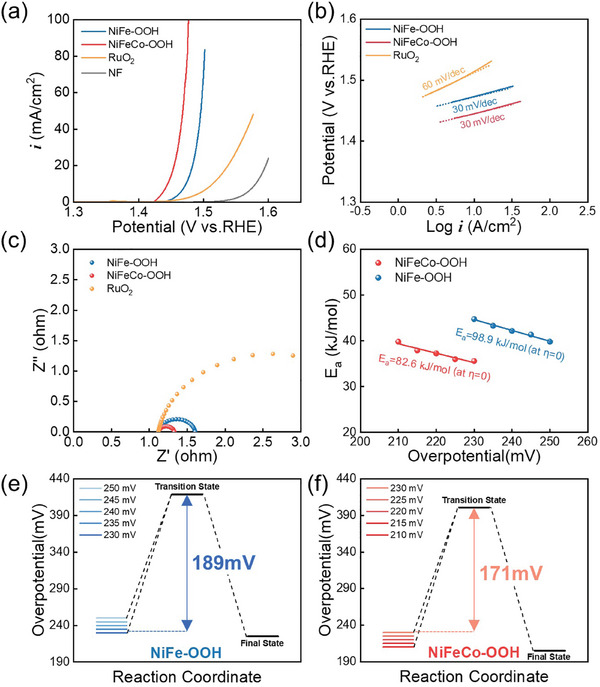
Electrochemical analysis of OER electrode. a) polarization curves of NF, RuO_2_, NiFe─OOH, and NiFeCo─OOH. b) Tafel plots. c) Electrochemical Impedance spectroscopy (EIS) at 1.53 V_RHE_. d) Variation of E_a_ as a function of η on NiFe─OOH and NiFeCo─OOH. e) Reaction paths of the rate‐determining step (RDS) in terms of η along a reaction coordinate on NiFe─OOH and NiFeCo─OOH.

XPS spectrums were obtained to investigate the electronic structure of NiFe─OOH and NiFeCo─OOH (Figure S5, Supporting Information). Figure 1f shows the high‐resolution XPS spectrum of Ni 2p. Upon the introduction of Co into NiFe─OOH, the binding energy of Ni 2p_3/2_ in NiFeCo─OOH shifted downward by 0.3 to 855.95 eV, indicating a significant interaction between Co and Ni. Figure 1g exhibits the high‐resolution XPS spectrum of Fe 2p. The binding energy of Fe 2p_3/2_ of NiFeCo─OOH and NiFe─OOH were similar at 711.6 eV. In contrast to Ni, the presence of Co did not affect the binding energy of Fe. Figure S6 (Supporting Information) shows the high‐resolution XPS spectrum of O 1s. Both NiFe─OOH and NiFeCo─OOH showed a strong peak at ≈531.5 eV, indicating hydroxyl species (OH).^[^ ^17^ ^]^ In addition, a shoulder peak corresponding to lattice oxygen species was observed at ≈530.0 eV for both samples.^[^ ^8a^ ^]^ Transition metal (oxy)hydroxides are known to possess both lattice oxygen species and hydroxyl species, and it has been confirmed that NiFe─OOH and NiFeCo─OOH contain both types of oxygen species.^[^ ^8^, ^18^ ^]^ The electrocatalytic OER activity of NiFe─OOH and NiFeCo─OOH was assessed in a 1m KOH solution using a three‐electrode system (**Figure** **2**a). For comparison, commercial RuO_2_ and nickel foam (NF) were tested under identical conditions. The OER activity was evaluated based on the overpotential required to achieve a current density of 10 mA cm^−2^. NF exhibited an overpotential of ≈351 mV, indicating poor OER activity. The overpotential for RuO_2_ coated on NF was ≈285 mV. Notably, NiFe─OOH showed a significantly lower overpotential (≈243 mV at 10 mA cm^−2^). Intriguingly, the introduction of Co into NiFe─OOH (NiFeCo─OOH) further enhanced the OER activity. NiFeCo─OOH demonstrated the lowest overpotential (≈214 mV at 10 mA cm^−2^), which was ≈29 mV lower than that of NiFe─OOH. Tafel plots were employed to investigate the kinetics of the OER (Figure 2b). The slope of RuO_2_ was ≈60 mV dec^−1^, indicating that the rate‐determining step (RDS) involved the coverage by the OH^−^ intermediate after the initial electron‐transfer step.^[^ ^19^ ^]^

The slopes of NiFe‐OOH and NiFeCo─OOH were ≈30 mV dec^−1^, indicating that the second electron transfer was the RDS.^[^ ^19a^ ^]^ The low Tafel slope indicates improved OER kinetics, implying that NiFe─OOH and NiFeCo─OOH exhibit superior OER kinetics compared to RuO_2_.^[^ ^20^ ^]^ Interestingly, it was confirmed that NiFeCo─OOH maintained the same Tafel slope (≈30 mV dec^−1^) as NiFe─OOH but exhibited a lower onset potential for the OER. Previous studies have reported that the addition of Co to NiFe─OOH shifts the onset of electrical conductivity to lower potentials while minimally affecting the intrinsic OER activity.^[^ ^21^ ^]^ Electrochemical surface areas (ECSA) were calculated by measuring the double‐layer capacitance (*C*_dl_), as shown in Figures S7 and S8 (Supporting Information).^[^ ^22^ ^]^ The ECSA calculated from *C*_dl_ was almost similar for both NiFe─OOH and NiFeCo─OOH because the surface morphology was similar. In addition, the LSV was normalized by ECSA to investigate the OER activity per ECSA (Figure S9, Supporting Information). Similarly, NiFeCo─OOH showed better OER activity per ECSA than NiFe─OOH. The charge transfer resistance (R_ct_) of RuO_2_, NiFe─OOH, and NiFeCo─OOH were measured by electrochemical impedance spectroscopy (EIS), as shown in Figure 2c. EIS results showed OER activity similar to that demonstrated with LSV. NiFeCo─OOH exhibited the lowest charge transfer resistance, indicating that NiFeCo─OOH has the best OER kinetics.^[^ ^23^ ^]^ In addition, the OER activity of NiFeCo─OOH was compared with that of a recently reported best‐performing hydroxide‐based OER electrocatalysts, and the NiFeCo─OOH showed comparable OER activity (Table S1, Supporting Information).

To investigate the effect of Co, the Arrhenius activation energy (E_a_) of NiFeCo─OOH and NiFe─OOH for OER was calculated.^[^ ^16^, ^24^ ^]^ Since the RDS for both NiFeCo─OOH and NiFe─OOH is determined by the second electron transfer (≈30 mV dec^−1^), the effect of Co can be confirmed by examining the kinetic energy barrier of this step. The activation energies were calculated using the Arrhenius equation.^[^ ^25^ ^]^ Figure S10 (Supporting Information) shows the LSV curves of NiFeCo─OOH and NiFe─OOH for OER at different temperatures, and the corresponding linear parts of the ln j versus overpotential at different temperatures are shown in Figure S11 (Supporting Information). The linearity between ln j and the overpotential indicates that the charge transfer reaction across the catalyst/solution interface is the RDS of OER. Figure S12 (Supporting Information) shows the Arrhenius plots of five selected overpotentials on the NiFeCo─OOH and NiFe─OOH. According to the Arrhenius equation, the slopes represent the activation energy. Then, the relationship between overpotential and E_a_ was plotted to quantify the dependence of the kinetic energy barrier on overpotential (Figure 2d). Since the activation energy at η = 0 mV represents the kinetic energy barrier at equilibrium, the values of *E*_a_ at η = 0 mV were obtained by extrapolation.^[^ ^24^, ^26^ ^]^ The values of *E*_a_ were 98.9 (NiFe─OOH) and 82.6kJ · mol^−1^ (NiFeCo─OOH), respectively. The lower activation energy of NiFeCo─OOH compared to NiFe─OOH suggests that the introduction of Co improved the OER kinetics by reducing the activation energy. In addition, the reaction paths of the RDS in terms of overpotential along a reaction coordinate were presented in Figure 2e,f. As the overpotential increased, the value of *E*_a_ decreased, and finally, the activation energy became zero. However, as the overpotential gradually increases, the RDS switches to a diffusion process rather than charge transfer. Therefore, the overpotential at *E*_a_ = 0 was calculated through extrapolation. The overpotentials at *E*_a_ = 0 were 419 mV (NiFe─OOH) and 401 mV (NiFeCo─OOH), indicating the transition states. As the overpotential increases, the kinetic energy barrier decreases. At the same overpotential of 230 mV, the kinetic energy barriers were 189 mV (NiFe─OOH) and 171 mV (NiFeCo─OOH). To evaluate the durability,^[^ ^27^ ^]^ NiFeCo─OOH was tested at 100mA cm^−2^ for 300 h (Figure S13, Supporting Information). It was observed that the change in potential was fairly stable despite being evaluated under harsh conditions. The chemical states of Ni and Fe were investigated using XPS analysis before/after OER. There was no significant difference in their chemical states before/after OER (Figure S14, Supporting Information). The presence of Co was confirmed through TEM‐EDS analysis following OER. Cobalt was observed to be uniformly distributed even after the OER, with a confirmed composition of ≈4.6 at.% (Figure S15, Supporting Information).

We investigated the effect of Co doping on the OER activity of NiFe─OOH using DFT calculations. Based on the X‐ray diffraction (XRD) results, we constructed an orthorhombic NiFe─OOH crystal system with a Pbnm space group that is consistent with XRD reference (JCPDS No. 01‐081‐0462).^[^ ^8^, ^28^ ^]^ Four unit cell structures with a Ni:Fe composition of 1:3 were examined, and the lowest energy unit cell was selected for further analysis. Based on the experimental results derived from XRD and SAED, we identified the (021) and (002) facets as potentially significant elements in NiFe─OOH catalyst systems for the OER. To determine a more stable surface for the OER mechanism in NiFe─OOH, we calculated the surface energies of the two identified facets.^[^ ^29^ ^]^ We found that the (021) facet of NiFe─OOH has a surface energy that was lower than the (002) facet by 0.013 eV cm^−2^. To identify the stable configuration of Co doping, we constructed eight slab models with different Co sites (Figure S16, Supporting Information). The relative stability of Co substitution was evaluated by calculating the formation energy (Δ*E_f_*) using the following equation:

(1) $$\Delta E_{f}=E_{slab}\;-\Sigma N_{i}E_{i}$$

where *E_slab_*, *E_i_*, and *N_i_* are the total energies of the slab model, the energy of the *i*‐th element, and the number of the *i*‐th element, respectively.^[^ ^30^ ^]^ Our calculated results show that the Co atom prefers to replace the Ni site at the surface, as this configuration exhibited the most negative Δ*E_f_* value. As a result, we were able to obtain the optimal active surface models of NiFe─OOH and NiFeCo─OOH for oxygen evolution reaction (OER) studies. In experimental observations, Co doping in NiFe─OOH showed a significant increase in catalytic activity. We aimed to validate the effect of Co through a theoretical approach. The Sabatier principle, which is proposed to elucidate reaction kinetics, states that the catalytic activity is governed by the relative adsorption energies of the OER intermediates.^[^ ^31^ ^]^ To calculate the OER activity, the following four‐electron reaction pathways were taken into account (Equations (2), (3), (4), (5)).

(2) $$\Delta G_{1}\;:\;4OH^{-}(\mathrm{aq})\;↔\;OH^{*}+\;3OH^{-}(\mathrm{aq})\;+\;e^{-}$$

(3) $$\begin{array}{cc} & \Delta G_{2}\;:\;OH^{*}\;+\;3OH^{-}\left(\mathrm{aq}\right)\;+\;e^{-}\\ & \;\;↔\;O^{*}+\;H_{2}O\left(l\right)\;+\;2OH^{-}(\mathrm{aq})\;+\;2e^{-} \end{array}$$

(4) $$\begin{array}{cc} & \Delta G_{3}\;:\;O^{*}\;+\;H_{2}O\left(l\right)+\;2OH^{-}\left(\mathrm{aq}\right)\;+\;2e^{-}\\ & \;\;↔\;\mathrm{OO}H^{*}+\;H_{2}O\left(l\right)\;+\;OH^{-}(\mathrm{aq})\;+\;3e^{-} \end{array}$$

(5) $$\begin{array}{cc} & \Delta G_{4}\;:\;\mathrm{OO}H^{*}+\;H_{2}O\left(l\right)\;+\;OH^{-}\left(\mathrm{aq}\right)\;+\;3e^{-}\\ & \;\;↔\;O_{2}\left(g\right)\;+\;2H_{2}O\left(l\right)\;+\;4e^{-} \end{array}$$

**Figure 3:**
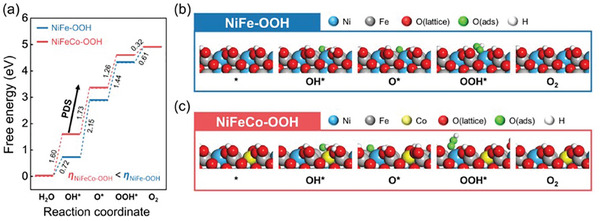
DFT calculation for Alkaline OER. a) Gibbs free energy diagram on NiFe─OOH and NiFeCo─OOH surfaces at U = 0 V. Most stable reaction intermediates on the b) NiFe─OOH and c) NiFeCo─OOH surfaces. Blue, gray, yellow, red, green, and white balls represent nickel, iron, cobalt, oxygen (lattice), oxygen (adsorbate), and hydrogen, respectively.

The overpotential (η) for OER was ascertained from the reaction energy diagram constructed based on the following equations (Equation (6), (7)). ^[^ ^32^ ^]^

(6) $$\Delta G\left(U\right)\;=\;\Delta E\;+\;\Delta \mathrm{ZPE}\;-\;T\Delta S\;-\;\mathrm{neU}$$

(7) $$\eta \;=\;U_{L}\;-\;1.23$$

where Δ*E* is the reaction energy in the ground state, Δ*ZPE* is the zero‐point energy correction, Δ*S* is the entropy difference, *U* is the applied potential, and *U*_L_ is the limiting potential.^[^ ^33^ ^]^ We considered three important reaction intermediates (O^*^, OH^*^, and OOH^*^) to construct a Gibbs free energy diagram for the OER mechanism. In this study, we investigated the OER mechanism on both NiFe─OOH (021) and NiFeCo─OOH (021). We derived their optimized structures with the reaction intermediates (O, OH, and OOH), and constructed Gibbs free‐energy diagrams at U = 0, 1.23 V, and their respective limiting potentials (Figure S17, Supporting Information). The potential‐determining step of NiFe─OOH and NiFeCo─OOH corresponded to the transformation of ^*^OH to ^*^O, with overpotentials of η = 920 and 500 mV, respectively (**Figure** **3**). The OER activity of NiFeCo─OOH (021) was higher than NiFe─OOH (021), in agreement with the experimental results.

**Figure 4:**
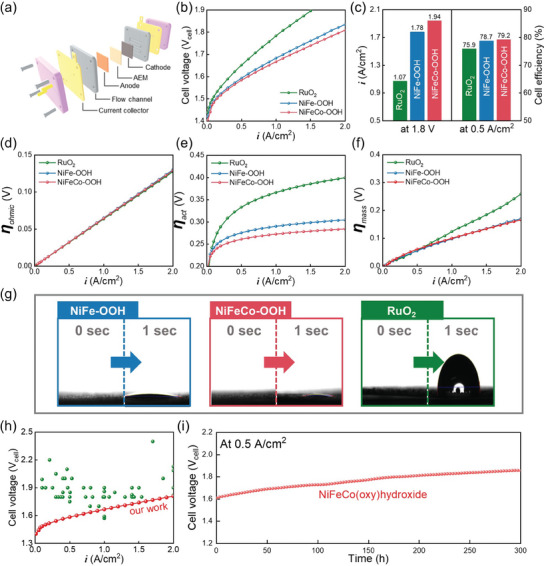
Test of AEM electrolyzers. a) Schematic of AEM electrolyzer. b) The polarization curves of AEM‐electrolyzers equipped with RuO_2_, NiFe─OOH, and NiFeCo─OOH. c) Comparison of current density at 1.8 V_cell_ and cell efficiency at 0.5 A cm^−2^. d) Ohmic loss of AEM electrolyzers. e) Activation loss of AEM electrolyzers. f) Mass transport loss of AEM electrolyzers. g) Wettability test of RuO_2_, NiFe─OOH, and NiFeCo─OOH. h) Performance comparison of AEM electrolyzers. i) Durability test of AEM electrolyzer at 0.5 A cm^−2^ for 300 h.

To investigate the applicability of NiFeCo─OOH in devices, AEM electrolyzers were assembled. The AEM electrolyzer comprises an anion exchange membrane (AEM), anode (OER electrode), cathode (HER electrode), a current collector, a graphite flow channel, a titanium flow channel, a graphite flow channel, and an end plate (**Figure** **4**a). To investigate the effect of the OER electrode on the AEM electrolyzer, Pt/C, the best electrocatalyst that can minimize polarization loss by HER, was used as the HER electrocatalyst.^[^ ^34^ ^]^ Figure 4b shows the polarization curves of the AEM electrolyzer equipped with RuO_2_, NiFe─OOH, and NiFeCo─OOH. The current density of the AEM electrolyzer at 1.8 V_cell_ was 1.07 A cm^−2^ (RuO_2_), 1.78 A cm^−2^ (NiFe─OOH), and 1.94 A cm^−2^ (NiFeCo─OOH), indicating that the AEM electrolyzer equipped with NiFeCo─OOH showed best performance (Figure 4c). In addition, the AEM electrolyzer equipped with NiFeCo─OOH required only 1.809 V_cell_ to deliver 2.0 A cm^−2^. The cell efficiency representing the energy conversion efficiency was calculated at a current density of 0.5 A cm^−2^, and the energy efficiency of NiFeCo─OOH was the highest at 79.2% (Figure 4c). In addition, it was confirmed that the cell efficiency of the AEM electrolyzer equipped with NiFeCo─OOH was high enough to satisfy the U.S. Department of Energy (DOE) target efficiency (77%), even at a current density of 0.7 A cm^−2^ (Figure S18, Supporting Information).^[^ ^35^ ^]^ To analyze the electrochemical behavior of the AEM electrolyzer according to the OER electrode, the overvoltage was divided into three resistances: ohmic overvoltage (η_ohm_), activation overvoltage (η_act_), and mass transport overvoltage (η_mass_), as shown in Figure S19 (Supporting Information).^[^ ^8^, ^36^ ^]^ The ohmic overvoltage of the AEM electrolyzer equipped with RuO_2_, NiFe─OOH, and NiFeCo─OOH was similar with increasing current density (Figure 4d). In general, the ohmic loss of the AEM electrolyzer is mainly determined by the AEM.^[^ ^37^ ^]^ Interestingly, the activation overvoltage of the AEM electrolyzer affected by the kinetics showed significant differences (Figure 4e). The kinetics of the AEM electrolyzer equipped with NiFeCo─OOH and NiFe─OOH outperformed the AEM electrolyzer equipped with RuO_2_. In addition, the AEM electrolyzer equipped with NiFeCo─OOH showed the best kinetics. The mass transport overvoltage of the AEM electrolyzer equipped with NiFe─OOH and NiFeCo─OOH was similar and much lower than that of RuO_2_(Figure 4f). Since the gas bubbles are vigorously generated at high current densities, efficient removal of the gases reduces the voltage losses due to mass transport.^[^ ^38^ ^]^ In general, the hydrophilic catalyst layer has an aerophobic property capable of quickly removing gases.^[^ ^38^, ^39^ ^]^ Even if water is dropped on the nickel foam and RuO_2_, the water does not permeate quickly into the nickel foam or catalyst layer (Figure S20, Supporting Information). However, NiFe‐OOH and NiFeCo─OOH are rapidly absorbed throughout the catalyst layer as soon as the droplets fall (Figure 4g). Therefore, AEM electrolyzers equipped with NiFe─OOH and NiFeCo─OOH showed low mass transfer loss even at high current densities. And, our AEM electrolyzer equipped with NiFeCo─OOH showed better performance than the AEM electrolyzers reported so far (Figure 4h; Table S2, Supporting Information).

A durability test was performed for 300 h at a current density of 0.5 A cm^−2^ (Figure 4i). There are two causes of cell deterioration. First, there is physical deterioration, which is attributed to physical damages caused by the electrolyte injected at a rate of 50 mL min^−1^ and detachment of the catalyst due to the violently generated gas. Second, chemical deterioration occurs as the electrochemical reaction progresses, leading to degradation of the separator, AEM, and electrode, thereby causing the voltage to rise. These two factors simultaneously contribute to cell degradation. Nevertheless, our AEM electrolyzer demonstrated a degradation rate of ≈900 µV h^−1^ over a 300‐h durability test. Compared to many studies reporting degradation rates of ≈1 mV h^−1^, our AEM electrolyzer shows relatively stable performance. In addition, it showed a high efficiency of >70% even after the end of the durability test (Figure S21, Supporting Information). The Faraday efficiency was calculated by collecting hydrogen generated during the endurance test and showed an efficiency of ≈99% (Figure S22, Supporting Information).

**Figure 5:**
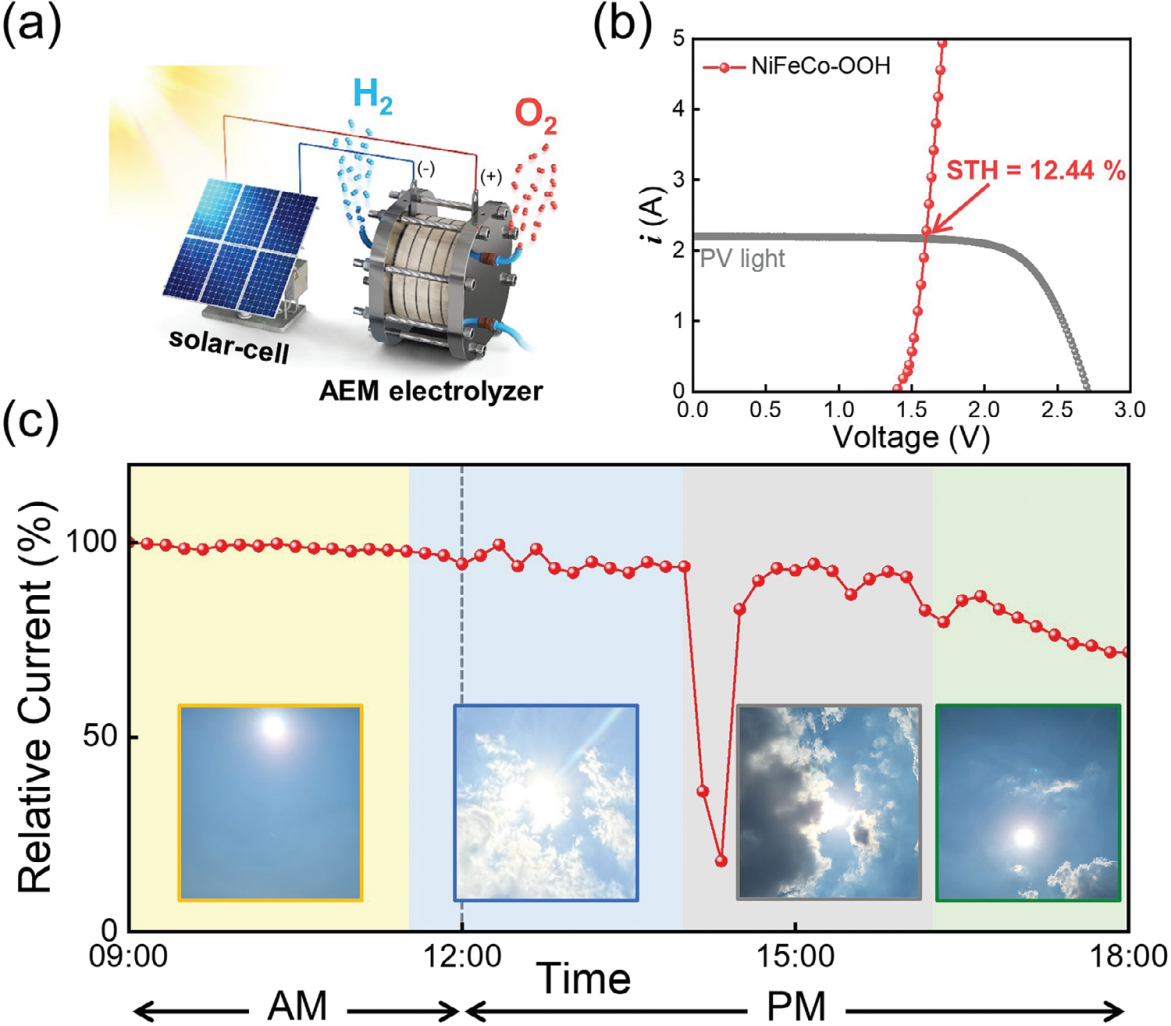
Solar‐powered AEM electrolyzer. a) schematic of an integrated system of solar cells and AEM electrolyzer. b) Current density–voltage (*J–V*) curves under dark and simulated AM 1.5G 100 mW cm^−2^ illumination for silicon solar cell combined with the AEM electrolyzer equipped with NiFeCo─OOH. c) Test the integrated system under natural lighting conditions.

By integrating the AEM electrolyzer with a commercially available silicon solar cell, the feasibility of producing green hydrogen without carbon emissions using renewable electricity was demonstrated (**Figure** **5**a). The AEM electrolyzer equipped with NiFeCo─OOH was connected to the silicon solar cell, and the polarization curve was measured under simulated AM 1.5G 100 mW cm^−2^ illumination after connecting 4‐silicon solar cells (Figure 5b). The commercial 4‐series solar module, of which total area is 212.48 cm^2^, showed a voltage of 2.182 V and current of 1.973 A in maximum power point (VMPP and IMPP) with one sun illumination (100 mW cm^−2^), and the PV‐EC operating current and voltage are 2.17 A and 1.594 V, respectively. The AEM electrolyzer was operated at 55 °C. To more accurately calculate the Solar‐to‐Hydrogen Efficiency (STH) of an integrated system of AEM electrolyzer and silicon solar cells, STH was recalculated taking into account the area of the solar cells.^[^ ^40^ ^]^ The integrated system showed a high solar‐to‐hydrogen (STH) efficiency of ≈12.44%. To evaluate the performance of the integrated system in an outdoor environment, an experiment was conducted from 09:00 AM to 06:00 PM (18:00) under natural lighting conditions (Figure 5c). Since outdoor conditions are not ideal (one sun),^[^ ^41^ ^]^ the integrated system exhibits relatively lower outputs, resulting in ≈10–20% lower current. From 9:00 AM to 12:00 PM, there were no clouds obstructing the sun, resulting in a consistent power output from the solar cells. As a result, the current of the AEM electrolyzer remained constant. However, from ≈12:00 to 2:00 PM, fluctuations in current were observed, indicating that the power generation was unstable due to clouds partially blocking the sunlight (inset image). Around 2:30 PM, the sun was completely obscured by clouds, resulting in a significant decrease in current from the AEM electrolyzer for a certain period of time (inset image). Fluctuations in current, as observed in renewable energy‐powered electrolyzers, are commonly encountered issues.^[^ ^42^ ^]^ These fluctuations are known to accelerate the degradation of electrolyzers.^[^ ^43^ ^]^ Furthermore, a gradual decrease in current was observed from 3:00 PM onward. This decrease can be attributed to the diminishing energy transfer from the sun as the solar elevation decreased. Hydrogen produced from renewable energy sources is a sustainable and environmentally friendly energy solution, and this study has presented, for the first time, the possibility of integrating solar cells and AEM electrolyzers for environmentally friendly hydrogen production. Furthermore, we aspire to achieve sustainable and cost‐effective hydrogen production through the development of an integrated system utilizing non‐precious metal‐driven AEM electrolyzers and solar cells.

## 3 Conclusion

In summary, we successfully developed NiFeCo─OOH as the OER electrocatalyst for the solar‐powered AEM electrolyzer. The incorporation of Co into NiFe─OOH resulted in improved OER activity, characterized by a reduced onset potential and activation energy while maintaining favorable OER kinetics. From DFT analysis, we were able to confirm that the incorporation of Co into NiFe─OOH led to an improvement in OER activity, a finding consistent with our experimental results. Consequently, the AEM electrolyzer equipped with NiFeCo─OOH demonstrated lower activation loss compared to NiFe─OOH, leading to remarkable performance. Specifically, the AEM electrolyzer equipped with NiFeCo─OOH achieved a current density of 2.0 A cm^−2^ at 1.809 V_cell_. Additionally, we constructed a system integrating the AEM electrolyzer with a silicon solar cell, which exhibited an impressive solar‐to‐hydrogen (STH) efficiency of ≈12.44%.

## 4 Experimental Section

### Preparation of NiFeCo─OOH and NiFe─OOH

NiFeCo─OOH was prepared by corrosion engineering. Commercial nickel foam (NF) was first immersed in 5 m HCl solution for 15 min to remove the surface oxide layer and then washed with deionized water (DI water). The prepared NF was immersed in a 25 mL solution containing 0.5 mm Co(NO_3_)_2_·6H_2_O and 2 mm Fe(NO_3_)_3_·9H_2_O. The corrosion process was carried out at room temperature for 24 h. The prepared sample was thoroughly rinsed with DI water and dried in a convection oven. This same was named as NiFeCo─OOH. For the preparation of NiFe─OOH, a 25 mL solution containing 2 mm Fe(NO_3_)_3_·9H_2_O was used and all other steps were identical to those for NiFeCo─OOH.

### Characterization of Physical Properties

The surface morphology of NiFeCo─OOH and NiFe‐OOH were observed using field emission scanning electron microscopy (FESEM, CZ/MIRAI LMH, TESCAN) equipped with an energy‐dispersive X‐ray spectrometer. High‐resolution transmission electron microscopy (HR‐TEM) images and selected area electron diffraction (SAED) patterns were recorded on a TALOS F200X (Thermo Fisher Scientific, USA) instrument. X‐ray diffraction patterns were recorded on an X‐ray diffractometer (D/MAX 2500, Rigaku, Japan) at a scan speed of 1° min^−1^. X‐ray photoelectron spectroscopy (XPS) was performed on a K‐Alpha (Thermo Fisher Scientific, Waltham, MA) spectrometer.

### Electrochemical Characterization

All electrochemical analyses were performed using a potentiostat (ZIVE BP2C, WonATech) in a three‐electrode system. The prepared electrode (active area = 1 cm × 1 cm) was used as the working electrode and the graphite rod was used as the counter electrode. Hg/HgO (1 M KOH) was used as the reference electrode. Polarization curves for OER were recorded in 1m KOH solution at a scan rate of 1 mV s^−1^ with *iR‐*correction. The electrolyte resistance was determined using the x‐intersection of the Nyquist plot. All resistances corresponding to the electrolyte resistance were fully corrected (100%). Electrochemical surface areas (ECSA) were calculated by measuring a double‐layer capacitance (C_dl_). The value of C_dl_ was measured by cyclic voltammetry (CV) with different scan rates in a non‐faradaic region. The ECSA was calculated using the following equation: ECSA = C_dl_/C_s_. The value of C_s_ means smooth plane capacitance for metal surfaces, which is 40 uF cm^−2^.^[^ ^22^ ^]^ Durability was tested at a constant current density of 100 mA cm^−2^ for 300 h. The electrochemical impedance spectroscopy (EIS) was obtained at 1.53 V (vs. RHE) from 100 kHz to 1 Hz with an amplitude of 10.0 mV. All potentials were converted to potentials versus the reversible hydrogen electrode (RHE) using the Nernst equation.^[^ ^44^ ^]^ An activation energy (E_a_) of electrocatalysts was calculated by measuring the polarization curve at different temperatures (35 to 65°C). For comparison with the PGM‐based OER electrocatalyst, a RuO_2_‐coated electrode was prepared on NF. The RuO_2_ electrode was prepared by dropping a RuO_2_ ink solution onto nickel foam. This ink solution consisted of RuO_2_, ethanol, and 5 wt.% Nafion solution.^[^ ^45^ ^]^

### AEM Electrolyzer

All electrochemical analyses of the AEM electrolyzer were performed using a potentiostat (ZIVE BP2C, WonATech) and a DC power supply (MK‐W102). The AEM electrolyzer consisted of the cathode, anode, graphite flow channel, titanium flow channel, anion exchange membrane (AEM, X37‐50 Grade T, Dioxide Materials), and end plates. The prepared OER electrode, that is, NiFeCo─OOH, NiFe─OOH, was directly used as the anode. The RuO_2_ electrode was prepared by spraying a RuO_2_ ink solution onto nickel foam. This ink solution consisted of RuO_2_, ethanol, and 5 wt.% ionomer solution. The cathode was prepared with Pt/C (40 wt.%, HISPEC 4000, Johnson Matthey) with a Nafion binder on carbon cloth with a microporous layer. The loading amount of Pt was ≈1.0 mg cm^−2^. A 1m KOH solution was used as the electrolyte and was supplied to the AEM electrolyzer at a rate of 50 mL min^−1^. The operating temperature of the AEM electrolyzer was ≈55 °C. The active area of the AEM electrolyzer was 3.8 cm^2^. Polarization curves of the AEM electrolyzer were obtained by measuring the cell voltage at fixed current densities. The durability of the AEM electrolyzer was tested at a constant current of 0.5 A cm^−2^ for 300 h. Cell efficiency was calculated as the following equation.^[^ ^12b^ ^]^

(8) $$\mathrm{Cell}\;\mathrm{efficiency}\;\;\left(\%\right)=\;\frac{H_{2}\;power\;out}{AEMWE\;power}\times 100\%$$

where $\Delta H_{H_{2,\;}\mathrm{LHV}\;}$ is the LHV reaction enthalpy for water electrolysis (241.8 kJ mol^−1^).^[^ ^46^ ^]^ $n_{H_{2},\;\mathrm{measured}}$ is the measured hydrogen production rate (mol s^−1^). The AEM electrolyzer power was calculated by multiplying the cell voltage and current density to calculate the cell efficiency. A lower heating value (LHV) was used as an H_2_ power out. Detailed information was presented in Table S3 (Supporting Information). The photovoltaic characteristics were measured using a solar simulator (Oriel 300, Newport Co.) and a source meter (Keithley 2400) under 100 mW cm^−2^ (standard recommended AM 1.5G irradiance, 1 sun). The integrated system, combining the AEM electrolyzer with the solar cell, was tested on the rooftop of the Engineering Building at Chungbuk National University in Korea. This specific location was selected for its clear, unobstructed access to sunlight, essential for the optimal performance of the solar cell component.

### Computational Details

The Vienna Ab initio Software Package (VASP) was used to perform Density functional theory (DFT) calculations, with the revised Perdew (Burke) Ernzerhof functionals.^[^ ^47^ ^]^ The projector augmented wave (PAW) method was employed with a plane wave energy of up to 520 eV to replace the interactions of core electrons. The electronic structure and mean force convergence criteria were set to 10–5 eV and 0.01 eV/Å, respectively. To account for correlation effects and reduce self‐interaction errors, we used the Hubbard U‐parameter (DFT + U), with optimized effective interaction parameters *U*_eff_ (*U*_eff_ = *U* − J) of 2.5, 3.5, and 2.7 eV for Ni, Fe, and Co elements in NiFe─OOH and NiFeCo─OOH, respectively.^[^ ^48^ ^]^ A 20 Å vacuum thickness along the z‐axis was employed to avoid surface interactions and deposited oxygen‐containing intermediates (O, OH, and OOH) on the (021) surfaces of NiFe─OOH and NiFeCo─OOH for free‐energy calculations. During geometry optimization, the two top layers were relaxed of the slab, and fixed the other two layers, with a dipole correction applied along the z‐direction.

## Conflict of Interest

The authors declare no conflict of interest.

## Author Contributions

J.S.H. and Y.P. contributed equally to this work. J.S.H. and Y.P. led the manuscript writing. J.S.H., S.H.L., and J‐Y.J. performed the electrochemical analysis. H.J. and B.K.K. analyzed the SEM and TEM images. S.H.P, I.T.K, C.K., and S.J.L. conducted the XPS analysis and a literature search on OER performance. Y.P., S.M.K., and S.C. performed the test of solar power AEM electrolyzer. S.M.C, B.K.K., and Y.S.P developed the intellectual concept and provided supervisory guidance on experiments, data interpretation, and manuscript refinement.

## Supporting information

Supporting Information

## Data Availability

The data that support the findings of this study are available from the corresponding author upon reasonable request.
