# Supplementary material for: Solar‐Powered AEM Electrolyzer via PGM‐Free (Oxy)hydroxide Anode with Solar to Hydrogen Conversion Efficiency of 12.44%
Source: Adv Sci (Weinh). 2024 Apr 24;11(25):2401782. doi: 10.1002/advs.202401782 (PMC11220676; doi:10.1002/advs.202401782)
Supplement: Supplementary file 1 — Supporting Information [file ADVS-11-2401782-s001.pdf]

ADVANCED SCIENCE
Open Access

Supporting Information

for *Adv. Sci.*, DOI 10.1002/advs.202401782

Solar-Powered AEM Electrolyzer via PGM-Free (Oxy)hydroxide Anode with Solar to Hydrogen Conversion Efficiency of 12.44%

Jun Seok Ha, Youngtae Park, Jae-Yeop Jeong, Seung Hun Lee, Sung Jun Lee, In Tae Kim, Seo Hyun Park, Hyunsoo Jin, Soo Min Kim, Suwon Choi, Chiho Kim, Sung Mook Choi, Bong Kyun Kang*, Hyuck Mo Lee* and Yoo Sei Park**

Supplementary Information

Solar-powered AEM electrolyzer via PGM-free (oxy)hydroxide anode with solar to hydrogen conversion efficiency of 12.44 %

Jun Seok Ha, Youngtae Park, Jae-Yeop Jeong, Seung Hun Lee, Sung Jun Lee, In Tae Kim, Seo Hyun Park, Hyunsoo Jin, Soo Min Kim, Suwon Choi, Chiho Kim, Sung Mook Choi*, Bong Kyun Kang*, Hyuck Mo Lee*, Yoo Sei Park*

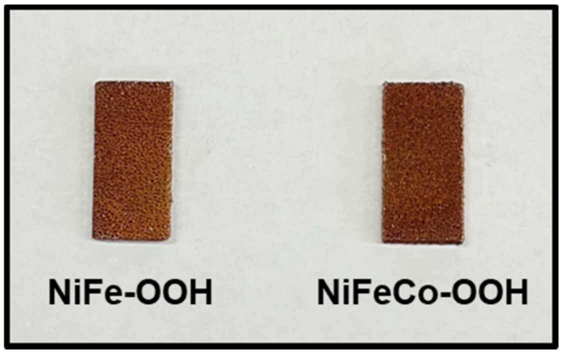

Figure S1. Photographs of NiFe-OOH and NiFeCo-OOH.

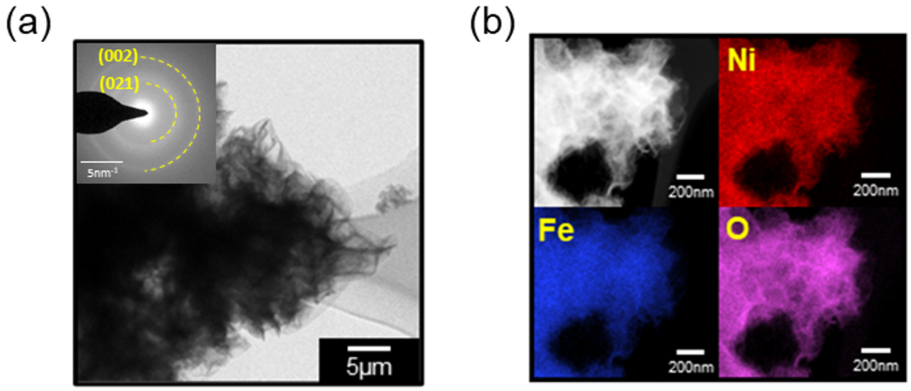

Figure S2. (a) TEM images of NiFe-OOH. (b) EDS mapping images of NiFe-OOH: Ni(red), Fe(blue), O(purple).

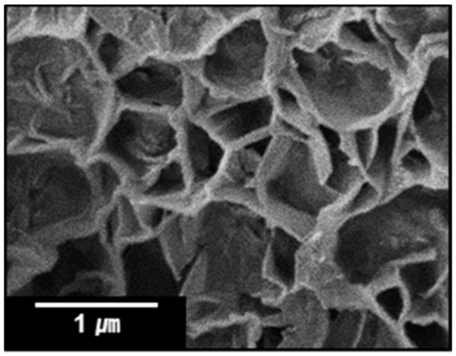

Figure S3. SEM images of NiFe-OOH.

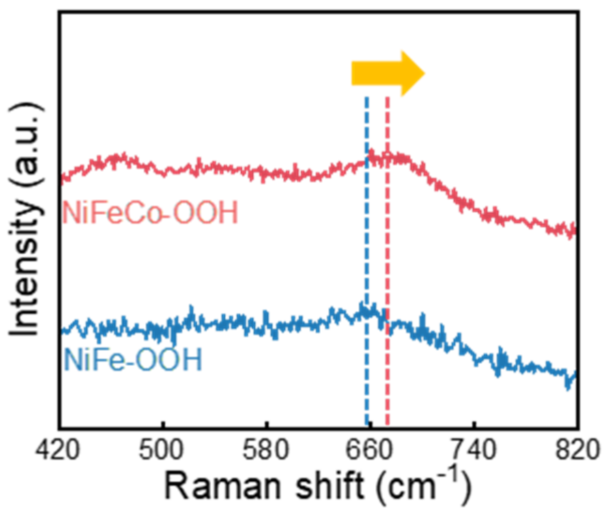

Figure S4. Raman spectra of the NiFe-OOH and NiFeCo-OOH.

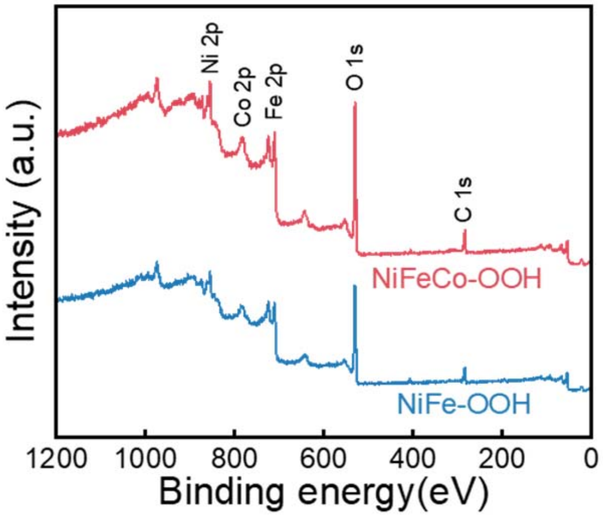

Figure S5. Full survey scan XPS spectrum of NiFe-OOH and NiFeCo-OOH.

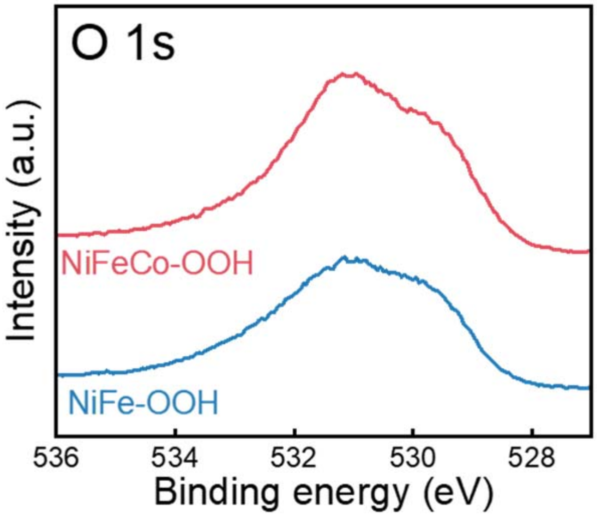

Figure S6. High-resolution XPS spectrum of O 1s.

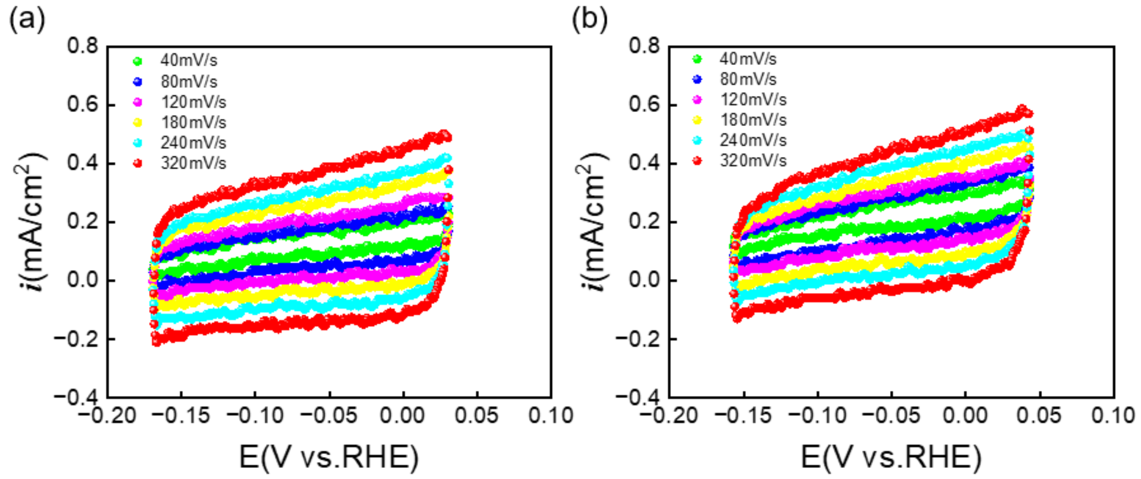

Figure S7. Cyclic voltammetry of NiFe-OOH and NiFeCo-OOH in non-faradaic region with different scan rates.

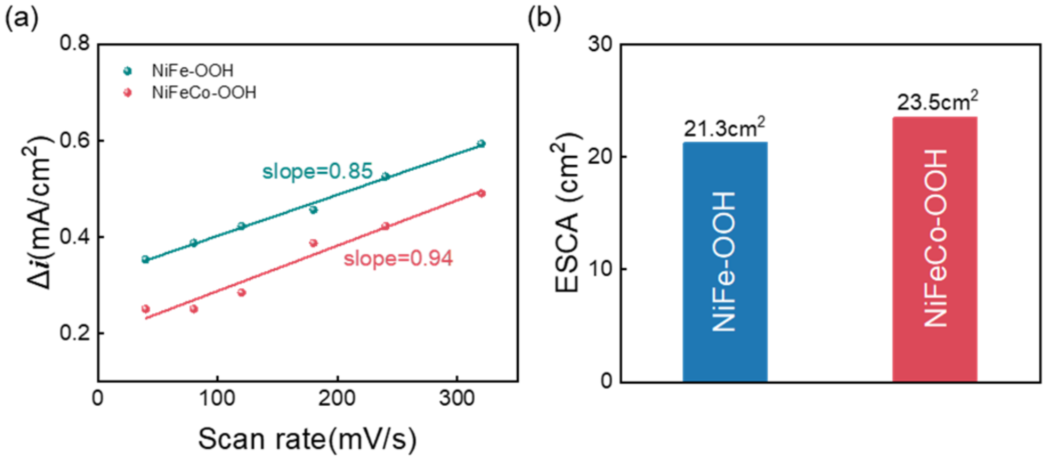

Figure S8. (a) Double layer capacitance (C_{dl}) of NiFe-OOH and NiFeCo-OOH. (b) Electrochemical surface areas of NiFe-OOH and NiFeCo-OOH.

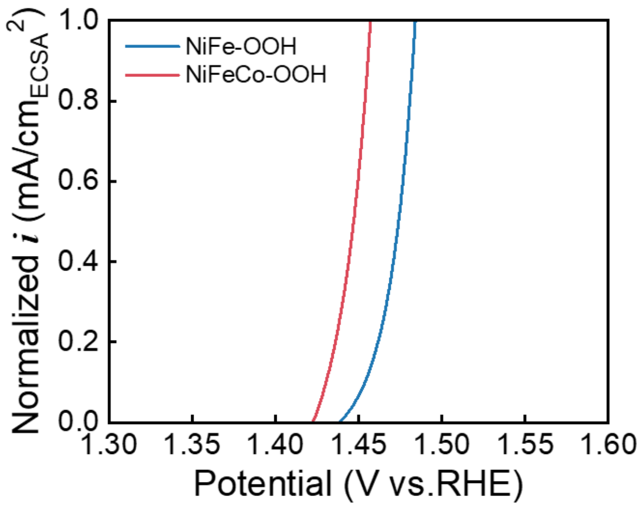

Figure S9. Polarization curves of NiFe-OOH and NiFeCo-OOH normalized by ECSA.

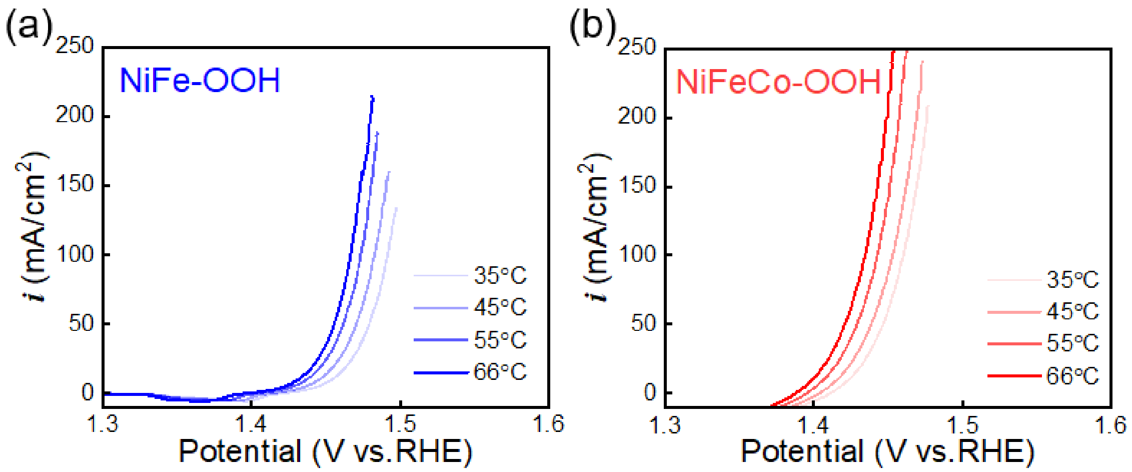

Figure S10. LSV curves of (a) NiFe-OOH and (b) NiFeCo-OOH at different temperatures.

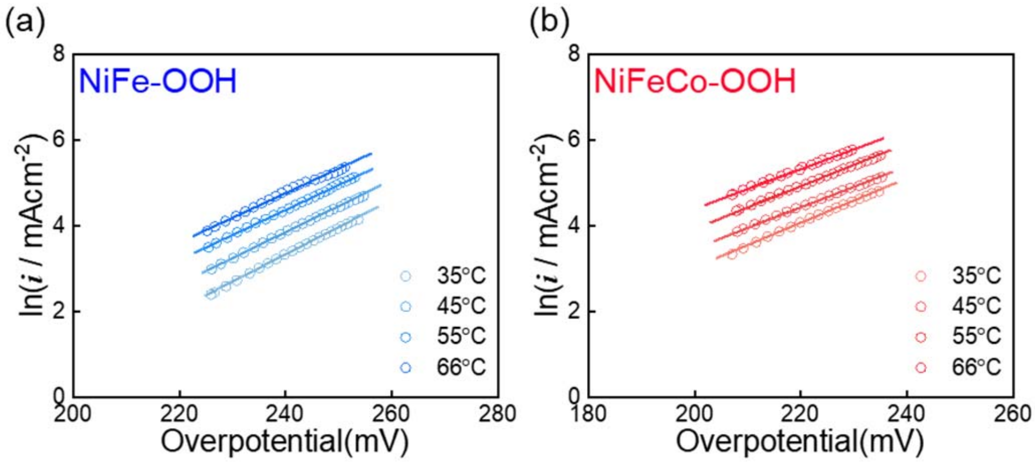

Figure S11. ln (***i*** / mAcm^{-2}) vs. overpotential plots of (a) NiFe-OOH, (b) NiFeCo-OOH at different temperatures.

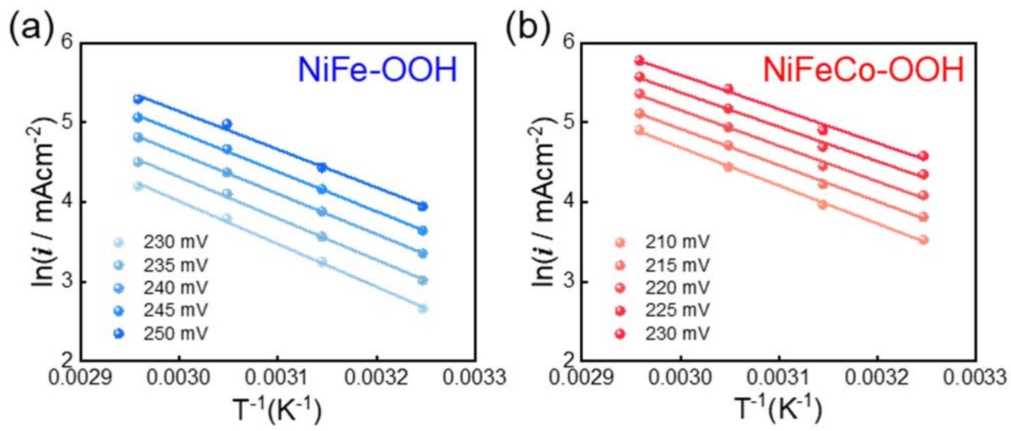

Figure S12. Arrhenius plots of (a) NiFe-OOH and (b) NiFeCo-OOH at different overpotentials.

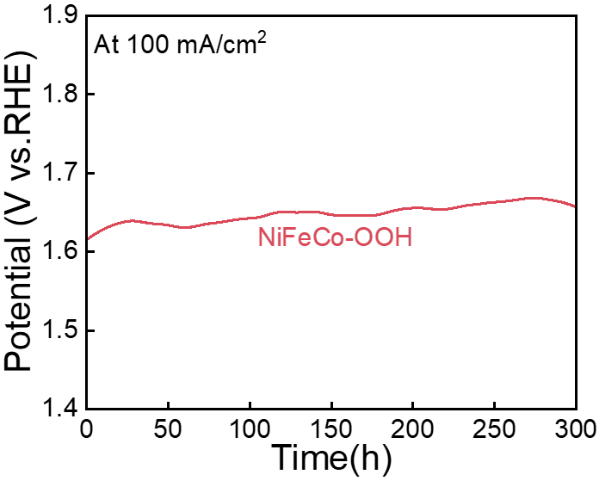

Figure S13. Durability test of NiFeCo-OOH at 100 mA/cm^2 for 300 h.

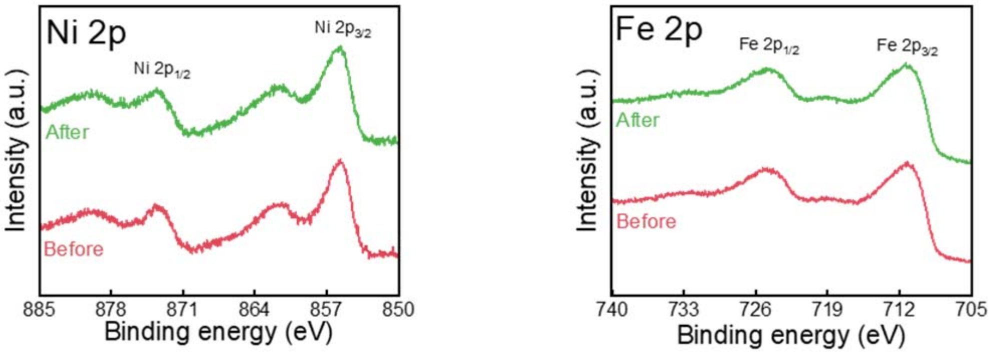

Figure S14. High-resolution XPS spectrum of Ni 2p and Fe 2p before/after OER.

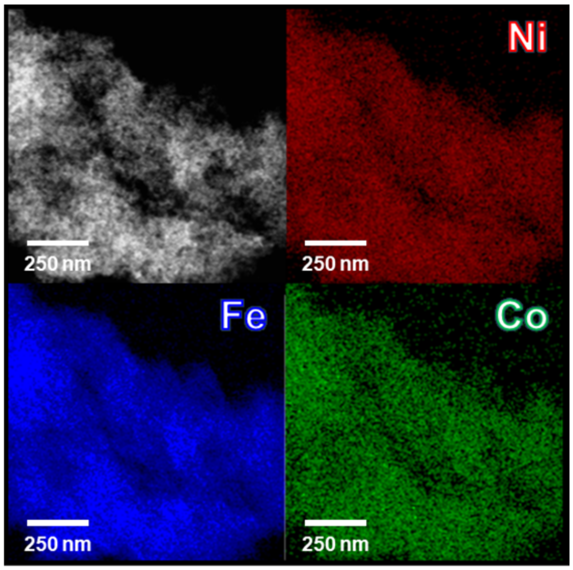

Figure S15. High-resolution XPS spectrum of Ni 2p and Fe 2p before/after OER.

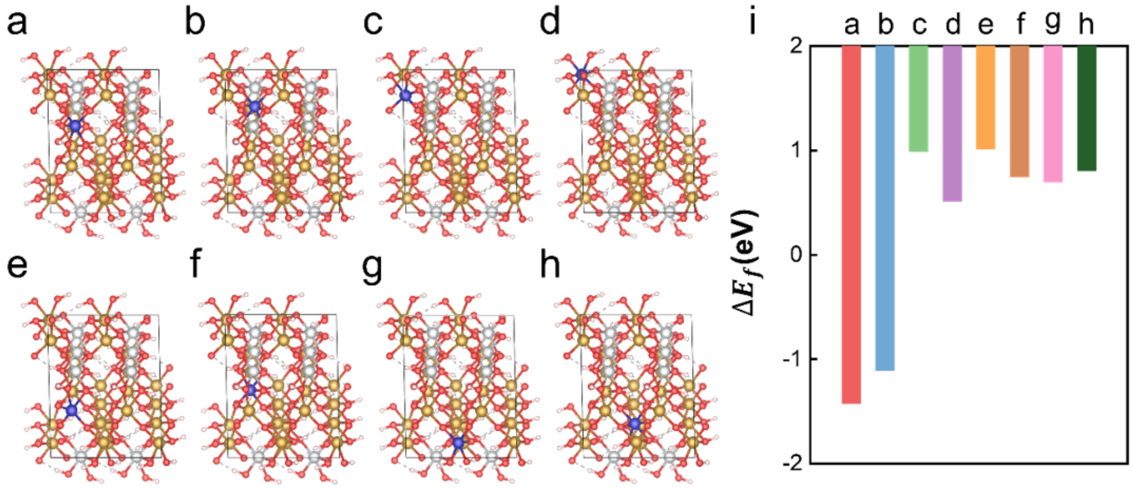

Figure S16. (a-h) Illustration of the eight slab models with different Co doping sites explored in this study. (i) The formation energy (ΔE_f) of each slab model.

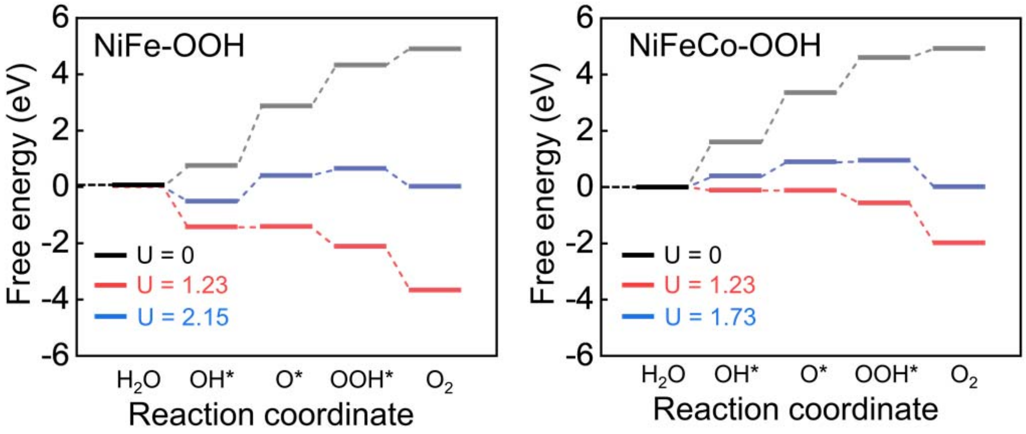

Figure S17. The free energy diagram of the (a) NiFe-OOH and (b) NiFeCo-OOH at U = 0 V, 1.23 V and their respective limiting potentials.

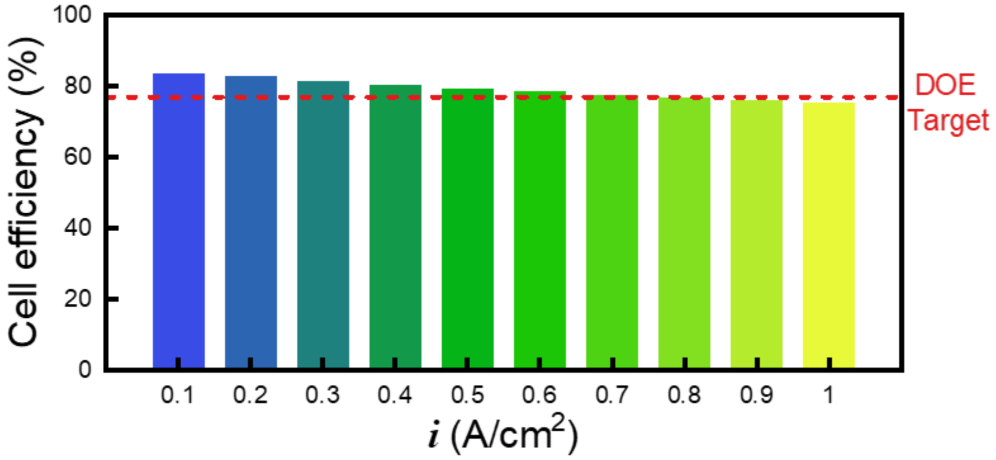

Figure S18. Cell efficiency of AEM electrolyzer equipped with NiFeCo-OOH at different current densities.

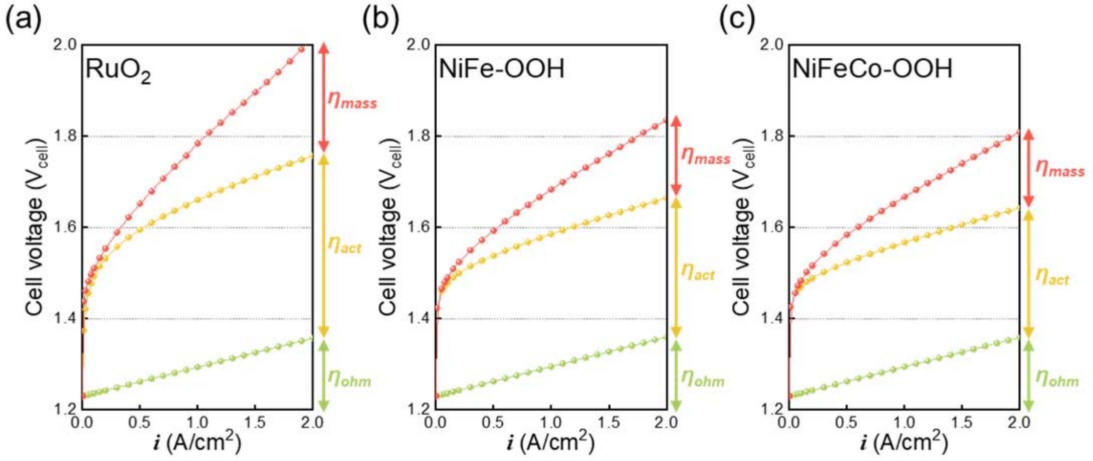

Figure S19. Deconvolution of cell voltage. (a) RuO_2. (b) NiFe-OOH. (c) NiFeCo-OOH.

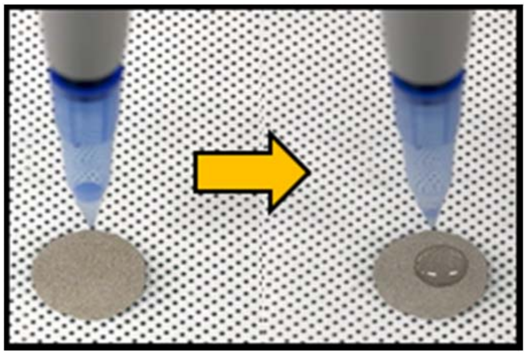

Figure S20. Photographs of wettability test of nickel foam.

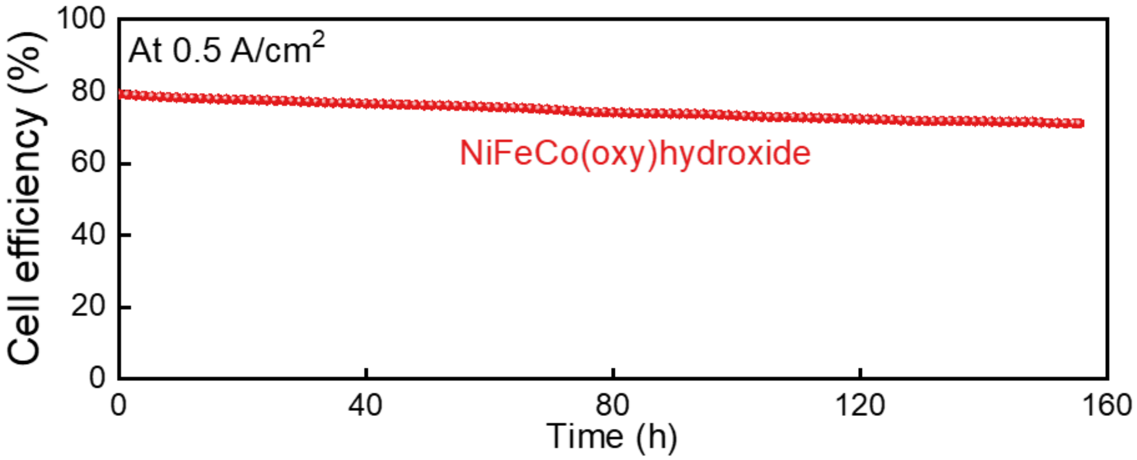

Figure S21. Faraday efficiency of AEM electrolyzer equipped with NiFeCo-OOH during the durability tests.

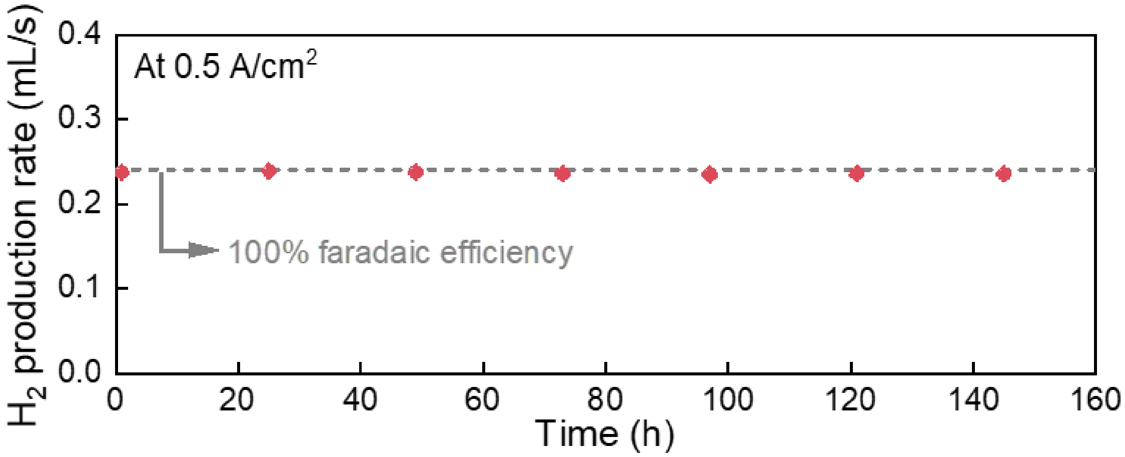

Figure S22. Faraday efficiency of AEM electrolyzer equipped with NiFeCo-OOH during the durability tests.

Table S1. Comparison of OER catalyst in alkaline solution (1M KOH)

Electrocatalysts	Overpotential (mV) at 10 mA/cm^2	Tafel slope (mV/dec)	Reference
NiFeCo-OOH	214	30	This work
NiFe-LDH0.4M HMS	290	51	1
NiFeCo-LDH/CF	249	42	2
MoNiFe-27% (oxy)hydroxide	242	23	3
CoMoIr-11%-NB	220	68	4
Co@NiFe-LDH	253	44	5
MOF-74 derived NiFe-LDH	299	48	6
CoNiN@NiFe-LDH	227	58	7
NiFe-LDH $Sn_{0.015(M)}$	250	66	8
$NiFeCoO_x$	248	32	9
NiFe-(Co32-)-LDH-1.5	228	37	10
NiFeCo-LDH/CF	220	58	11
Re-NiFeP NBs	246	41	12
CoP/FeOOH	290	56	13
NiFeOOH	209	32	14
S-NiFeOOH	220	44	15
FeOOH(Se)/IF	287	54	16
FeOOH/Cr-$NiCo_2O_4$/NF	217	31	17
FeNiOOH/FNF	252	37	18
Anodized FeNiCo	260	42	19
$Ni_{0.5}Fe_{0.5}/C$	270	40	20

Table S2. Performance comparison of AEM electrolyzer

Cathdoe	Anode	Temperature (℃)	Performance	Reference
Pt/C	NiFeCo-OOH	55	1.94 A/cm^2 at 1.8 V_{cell}	This work
NiFeCoP	NiFeCo-LDH	50	0.5 A/cm^2 at 1.75 V_{cell}	21
Pt/C	NiFeV-LDH	50	2.1 A/cm^2 at 1.8 V_{cell}	22
Pt/C	NiFeOOH	50	0.92 A/cm^2 at 1.7 V_{cell}	23
Pt/C	$Ni_{0.75}Fe_{2.25}O_4$	50	2 A/cm^2 at 1.9 V_{cell}	24
NiCu	Ir	50	1.85 A/cm^2 at 2 V_{cell}	25
RuP_2	IrO_2	50	1 A/cm^2 at 1.86 V_{cell}	26
Pt/C	$CoSb_2O_6$	60	0.8 A/cm^2 at 1.9 V_{cell}	27
NiCo/NiCoO	$CuCo_2O_4$	50	0.5 A/cm^2 at 1.85 V_{cell}	28
Pt/C	NiFe-LDH	50	1 A/cm^2 at 1.67 V_{cell}	29
CuCoP	IrO_2	50	0.7 A/cm^2 at 1.9 V_{cell}	30
NiCoS	IrO_2	50	1.7 A/cm^2 at 2.4 V_{cell}	31
$MoNi_4/MoO_2$	NiFe	70	1.15 A/cm^2 at 1.85 V_{cell}	32
Pt/C	$CuCo_2O_4$	50	1.3 A/cm^2 at 1.8 V_{cell}	33
Pt/C	IrO_2	60	2 A/cm^2 at 1.82 V_{cell}	34
Pt/C	NiFe-LDH	60	1 A/cm^2 at 1.67 V_{cell}	35
Co_3S_4	$Cu_{0.81}Co_{2.19}O_4$	60	0.431 A/cm^2 at 2 V_{cell}	36
Pt/C	$CuCo_2O_4$	60	1.4 A/cm^2 at 1.9 V_{cell}	37
Pt/C	$CuCo_2O_4$	60	1.39 A/cm^2 at 1.8 V_{cell}	38
Pt/C	CoFe	80	0.794 A/cm^2 at 1.7 V_{cell}	39
Fe-NiMoNH$_3$/H$_2$	NiMo-NH$_3$/H$_2$	80	1 A/cm^2 at 1.57 V_{cell}	40
Pt/C	$Cu_{0.95}Co_{2.05}O_4$	50	1.54 A/cm^2 at 1.8 V_{cell}	41
PtNi	Co	80	0.73 A/cm^2 at 1.8 V_{cell}	42
Pt/C	IrO_2	90	0.983 A/cm^2 at 1.8 V_{cell}	43
NiFeCo	$NiFeO_4$	60	2 A/cm^2 at 2.13 V_{cell}	44
Pt/C	$NiMn_2O_4$	50	0.53 A/cm^2 at 2 V_{cell}	45
NiAlMo	NiAlMo	60	2 A/cm^2 at 2.086 V_{cell}	46
Pt/C	NiFe	50	1.4 A/cm^2 at 1.823 V_{cell}	47
Pt/C	IrO_2	70	1.5 A/cm^2 at 1.9 V_{cell}	48
Pt/C	IrO_2	50	0.4 A/cm^2 at 2 V_{cell}	49
Pt/C	$CuCo_2O_4$	30	0.37 A/cm^2 at 1.8 V_{cell}	50
NiMo/C	Ir	50	1 A/cm^2 at 1.9 V_{cell}	51
Pt/C	IrO_2	50	1.07 A/cm^2 at 1.8 V_{cell}	52

NiFeCo	$NiFe_2O_4$	60	1 A/cm^2 at 1.9 V_{cell}	53
Pt/C	IrO_2	50	0.299 A/cm^2 at 1.8 V_{cell}	54
PtNi	PtNi	50	0.25 A/cm^2 at 1.9 V_{cell}	55
CoP	CoP	50	0.335 A/cm^2 at 1.8 V_{cell}	56
Ni/CeO_2-La_2O_3/C	$CuCoO_x$	70	0.208 A/cm^2 at 2.2 V_{cell}	57
Pt	$Pb_2Ru_2O_{6.5}$	50	0.5 A/cm^2 at 1.8 V_{cell}	58
Ni	$Ce_{0.2}MnFe_{1.8}O_4$	25	0.3 A/cm^2 at 1.8 V_{cell}	59
Ni/CeO_2-La_2O_3/C	$CuCoO_x$	43	0.47 A/cm^2 at 1.9 V_{cell}	60
Ni	Ni	50	0.15 A/cm^2 at 1.9 V_{cell}	61
Ni	$Li_{0.21}Co_{2.79}O_4$	45	0.3 A/cm^2 at 2.05 V_{cell}	62
Ni	$Cu_{0.7}Co_{2.3}O_4$	55	0.1 A/cm^2 at 1.99 V_{cell}	63
Pt/C	LDPE	45	0.46 A/cm^2 at 2.1 V_{cell}	64
Pt/C	IrO_2	50	0.399 A/cm^2 at 1.8 V_{cell}	65
NiMo	NiFe	40	0.4 A/cm^2 at 1.85 V_{cell}	66
Ni	$Cu_{0.7}Co_{2.3}O_4$	22	0.1 A/cm^2 at 1.9 V_{cell}	67
Pt/C	$Cu_{0.7}Co_{2.5}O_4$	25	1 A/cm^2 at 1.8 V_{cell}	68

Table S3. Calculation of cell efficiency.

The lower heating value (LHV) of H_2 was used to calculate the efficiency of MEA.

• H_2 production per second at 1.5 A/cm^2

$= (j\ A/cm^2) \times (1\ e^-/1.602 \times 10^{-19}\ C) \times (1\ H_2/2e^-)$

$= 1.5\ A/cm^2 / (1.602 \times 10^{-19}\ C \times 2)$

$= 4.682 \times 10^{18}\ H_2/cm^2 \times s$

$= 7.775 \times 10^{-6}\ mol \times H_2\ cm^{-2}\ s^{-1}$

• LHV for H_2

$= 120\ kJ/g\ H_2$

$= 2.42 \times 10^5\ J/mol\ H_2$

• H_2 power out

$= (7.775 \times 10^{-6}\ mol \times H_2/cm^2 \times s) \times (2.42 \times 10^5\ J/mol\ H_2)$

$= 1.88\ W/cm^2$

• AEMWE power

= Current density (A/cm^2) x Cell voltage (V)

• Cell efficiency

$$\text{Cell efficiency}(\%) = \frac{H_2\ power\ out}{AEMWE\ power} \times 100\%$$

References

[1] H. Zhong, T. Liu, S. Zhang, D. Li, P. Tang, N. Alonso-Vante, Y. Feng, Template-free synthesis of three-dimensional NiFe-LDH hollow microsphere with enhanced OER performance in alkaline media, Journal of Energy Chemistry, 33 (2019) 130-137.

[2] Y. Lin, H. Wang, C.-K. Peng, L. Bu, C.-L. Chiang, K. Tian, Y. Zhao, J. Zhao, Y.-G. Lin, J.-M. Lee, L. Gao, Co-Induced Electronic Optimization of Hierarchical NiFe LDH for Oxygen Evolution, Small, 16 (2020) 2002426.

[3] Z. He, J. Zhang, Z. Gong, H. Lei, D. Zhou, N. Zhang, W. Mai, S. Zhao, Y. Chen, Activating lattice oxygen in NiFe-based (oxy)hydroxide for water electrolysis, Nature Communications, 13 (2022) 2191.

[4] R. Jia, M. Xia, L. Tang, L. Yu, Y. Yang, Y. Zhang, X. Bo, S. Zhou, Y. Tu, D. Deng, Single-Atomic Ir and Mo Co-Confined in a Co Layered Hydroxide Nanobox Mutually Boost Oxygen Evolution, ACS Catalysis, 12 (2022) 13513-13522.

[5] S. Liu, R. Wan, Z. Lin, Z. Liu, Y. Liu, Y. Tian, D.-D. Qin, Z. Tang, Probing the Co role in promoting the OER and Zn–air battery performance of NiFe-LDH: a combined experimental and theoretical study, Journal of Materials Chemistry A, 10 (2022) 5244-5254.

[6] M. Rinawati, Y.-X. Wang, K.-Y. Chen, M.-H. Yeh, Designing a spontaneously deriving NiFe-LDH from bimetallic MOF-74 as an electrocatalyst for oxygen evolution reaction in alkaline solution, Chemical Engineering Journal, 423 (2021) 130204.

[7] J. Wang, G. Lv, C. Wang, A highly efficient and robust hybrid structure of CoNiN@NiFe LDH for overall water splitting by accelerating hydrogen evolution kinetics on NiFe LDH, Applied Surface Science, 570 (2021) 151182.

[8] K. Bera, R. Madhu, H.N. Dhandapani, S. Nagappan, A. De, S. Kundu, Accelerating the Electrocatalytic Performance of NiFe–LDH via Sn Doping toward the Water Oxidation Reaction under Alkaline Condition, Inorganic Chemistry, 61 (2022) 16895-16904.

[9] A.I. Inamdar, H.S. Chavan, S.M. Pawar, H. Kim, H. Im, NiFeCo oxide as an efficient and sustainable catalyst for the oxygen evolution reaction, International Journal of Energy Research, 44 (2020) 1789-1797.

[10] S. Liang, B. Wei, M. Yuan, Y. Li, X. Ma, Y. Wu, L. Xu, Self-supported Reevesite Ni-Fe Layered Double Hydroxide Nanosheet Arrays for Efficient Water Oxidation, ChemistrySelect, 5 (2020) 3062-3068.

[11] R. Hu, H. Jiang, J. Xian, S. Mi, L. Wei, G. Fang, J. Guo, S. Xu, Z. Liu, H. Jin, H. Yu, J. Wan, Pearson’s Principle-Inspired Robust 2D Amorphous Ni-Fe-Co Ternary

Hydroxides on Carbon Textile for High-Performance Electrocatalytic Water Splitting, Nanomaterials, 2022.
[12] P. Yan, Q. Liu, H. Zhang, L. Qiu, H.B. Wu, X.-Y. Yu, Deeply reconstructed hierarchical and defective NiOOH/FeOOH nanoboxes with accelerated kinetics for the oxygen evolution reaction, Journal of Materials Chemistry A, 9 (2021) 15586-15594.
[13] J. Cheng, B. Shen, Y. Song, J. Liu, Q. Ye, M. Mao, Y. Cheng, FeOOH decorated CoP porous nanofiber for enhanced oxygen evolution activity, Chemical Engineering Journal, 428 (2022) 131130.
[14] C. Jia, C. Zhen, L. Yin, H. Zhu, P. Du, A. Han, G. Liu, H.-M. Cheng, Topologic transition-induced abundant undercoordinated Fe active sites in NiFeOOH for superior oxygen evolution, Nano Energy, 106 (2023) 108044.
[15] C. Kim, S.H. Kim, S. Lee, I. Kwon, S.H. Kim, S. Kim, C. Seok, Y.S. Park, Y. Kim, Boosting overall water splitting by incorporating sulfur into NiFe (oxy)hydroxide, Journal of Energy Chemistry, 64 (2022) 364-371.
[16] S. Niu, W.-J. Jiang, Z. Wei, T. Tang, J. Ma, J.-S. Hu, L.-J. Wan, Se-Doping Activates FeOOH for Cost-Effective and Efficient Electrochemical Water Oxidation, Journal of the American Chemical Society, 141 (2019) 7005-7013.
[17] T. Liu, P. Diao, Nickel foam supported Cr-doped NiCo2O4/FeOOH nanoneedle arrays as a high-performance bifunctional electrocatalyst for overall water splitting, Nano Research, 13 (2020) 3299-3309.
[18] Z. Wang, Q. Lei, Z. Wang, H. Yuan, L. Cao, N. Qin, Z. Lu, J. Xiao, J. Liu, In-situ synthesis of free-standing FeNi-oxyhydroxide nanosheets as a highly efficient electrocatalyst for water oxidation, Chemical Engineering Journal, 395 (2020) 125180.
[19] M. Nishimoto, S. Kitano, D. Kowalski, Y. Aoki, H. Habazaki, Highly Active and Durable FeNiCo Oxyhydroxide Oxygen Evolution Reaction Electrocatalysts Derived from Fluoride Precursors, ACS Sustainable Chemistry & Engineering, 9 (2021) 9465-9473.
[20] Z. Wang, Y. Wang, N. Zhang, L. Ma, J. Sun, C. Yu, S. Liu, R. Jiang, Highly efficient oxygen evolution catalysis achieved by NiFe oxyhydroxide clusters anchored on carbon black, Journal of Materials Chemistry A, 10 (2022) 10342-10349.
[21]J. Lee, H. Jung, Y. S. Park, N. Kwon, S. Woo, N. C. S. Selvam, G. S. Han, H. S. Jung, P. J. Yoo, S. M. Choi, J. W. Han and B. Lim, Applied Catalysis B: Environmental, 2021, 294, 120246.
[22]J. Lee, H. Jung, Y. S. Park, S. Woo, J. Yang, M. J. Jang, J. Jeong, N. Kwon, B. Lim, J. W. Han and S. M. Choi, Small, 2021, 17, 2100639.

[23]Y. S. Park, J. Lee, M. J. Jang, J. Yang, J. Jeong, J. Park, Y. Kim, M. H. Seo, Z. Chen and S. M. Choi, Journal of Materials Chemistry A, 2021, 9, 9586-9592.
[24]J. Lee, H. Jung, Y. S. Park, S. Woo, N. Kwon, Y. Xing, S. H. Oh, S. M. Choi, J. W. Han and B. Lim, Chemical Engineering Journal, 2021, 420, 127670.
[25]A. Y. Faid, A. O. Barnett, F. Seland and S. Sunde, Electrochimica Acta, 2021, 371, 137837.
[26]J.-C. Kim, J. Kim, J. C. Park, S. H. Ahn and D.-W. Kim, Chemical Engineering Journal, 2021, 420, 130491.
[27]K. Ham, S. Hong, S. Kang, K. Cho and J. Lee, ACS Energy Letters, 2021, 6, 364-370.
[28]Y. S. Park, J. Jeong, Y. Noh, M. J. Jang, J. Lee, K. H. Lee, D. C. Lim, M. H. Seo, W. B. Kim, J. Yang and S. M. Choi, Applied Catalysis B: Environmental, 2021, 292, 120170.
[29]S. S. Jeon, J. Lim, P. W. Kang, J. W. Lee, G. Kang and H. Lee, ACS Applied Materials & Interfaces, 2021, 13, 37179-37186.
[30]W. Guo, J. Kim, H. Kim and S. H. Ahn, International Journal of Hydrogen Energy, 2021, 46, 19789-19801.
[31]W. Guo, J. Kim, H. Kim and S. H. Ahn, International Journal of Energy Research, 2021, 45, 1918-1931.
[32]P. Thangavel, M. Ha, S. Kumaraguru, A. Meena, A. N. Singh, A. M. Harzandi and K. S. Kim, Energy & Environmental Science, 2020, 13, 3447-3458.
[33]M. J. Jang, J. Yang, J. Lee, Y. S. Park, J. Jeong, S. M. Park, J.-Y. Jeong, Y. Yin, M.-H. Seo, S. M. Choi and K. H. Lee, Journal of Materials Chemistry A, 2020, 8, 4290-4299.
[34]P. Fortin, T. Khoza, X. Cao, S. Y. Martinsen, A. Oyarce Barnett and S. Holdcroft, Journal of Power Sources, 2020, 451, 227814.
[35]H. Koshikawa, H. Murase, T. Hayashi, K. Nakajima, H. Mashiko, S. Shiraishi and Y. Tsuji, ACS Catalysis, 2020, 10, 1886-1893.
[36]Y. S. Park, J. H. Lee, M. J. Jang, J. Jeong, S. M. Park, W.-S. Choi, Y. Kim, J. Yang and S. M. Choi, International Journal of Hydrogen Energy, 2020, 45, 36-45.
[37]Y. S. Park, M. J. Jang, J. Jeong, S. M. Park, X. Wang, M. H. Seo, S. M. Choi and J. Yang, ACS Sustainable Chemistry & Engineering, 2020, 8, 2344-2349.
[38]Y. S. Park, J. Yang, J. Lee, M. J. Jang, J. Jeong, W.-S. Choi, Y. Kim, Y. Yin, M. H. Seo, Z. Chen and S. M. Choi, Applied Catalysis B: Environmental, 2020, 278, 119276.
[39]S. Kang, K. Ham and J. Lee, Electrochimica Acta, 2020, 353, 136521.
[40]P. Chen and X. Hu, Advanced Energy Materials, 2020, 10, 2002285.

[41]C. Y. Kwon, J. Y. Jeong, J. Yang, Y. S. Park, J. Jeong, H. Park, Y. Kim and S. M. Choi, Front Chem, 2020, 8, 600908.
[42]S. M. Alia, M.-A. Ha, C. Ngo, G. C. Anderson, S. Ghoshal and S. Pylypenko, ACS Catalysis, 2020, 10, 9953-9966.
[43]A. Lim, H.-j. Kim, D. Henkensmeier, S. Jong Yoo, J. Young Kim, S. Young Lee, Y.-E. Sung, J. H. Jang and H. S. Park, Journal of Industrial and Engineering Chemistry, 2019, 76, 410-418.
[44]I. V. Pushkareva, A. S. Pushkarev, S. A. Grigoriev, P. Modisha and D. G. Bessarabov, International Journal of Hydrogen Energy, 2020, 45, 26070-26079.
[45]A. Carbone, S. C. Zignani, I. Gatto, S. Trocino and A. S. Aricò, International Journal of Hydrogen Energy, 2020, 45, 9285-9292.
[46]L. Wang, T. Weissbach, R. Reissner, A. Ansar, A. S. Gago, S. Holdcroft and K. A. Friedrich, ACS Applied Energy Materials, 2019, 2, 7903-7912.
[47]E. Cossar, A. Oyarce Barnett, F. Seland and E. A. Baranova, Catalysts, 2019, 9, 814.
[48]J. E. Park, S. Y. Kang, S.-H. Oh, J. K. Kim, M. S. Lim, C.-Y. Ahn, Y.-H. Cho and Y.-E. Sung, Electrochimica Acta, 2019, 295, 99-106.
[49]X. Su, L. Gao, L. Hu, N. A. Qaisrani, X. Yan, W. Zhang, X. Jiang, X. Ruan and G. He, Journal of Membrane Science, 2019, 581, 283-292.
[50]W.-S. Choi, M. J. Jang, Y. S. Park, K. H. Lee, J. Y. Lee, M.-H. Seo and S. M. Choi, ACS Applied Materials & Interfaces, 2018, 10, 38663-38668.
[51]A. Y. Faid, A. Oyarce Barnett, F. Seland and S. Sunde, Catalysts, 2018, 8, 614.
[52]M. K. Cho, H.-Y. Park, H. J. Lee, H.-J. Kim, A. Lim, D. Henkensmeier, S. J. Yoo, J. Y. Kim, S. Y. Lee, H. S. Park and J. H. Jang, Journal of Power Sources, 2018, 382, 22-29.
[53]J. J. Kaczur, H. Yang, Z. Liu, S. D. Sajjad and R. I. Masel, Frontiers in Chemistry, 2018, 6.
[54]M. K. Cho, H.-Y. Park, S. Choe, S. J. Yoo, J. Y. Kim, H.-J. Kim, D. Henkensmeier, S. Y. Lee, Y.-E. Sung, H. S. Park and J. H. Jang, Journal of Power Sources, 2017, 347, 283-290.
[55]S. H. Ahn, S. J. Yoo, H.-J. Kim, D. Henkensmeier, S. W. Nam, S.-K. Kim and J. H. Jang, Applied Catalysis B: Environmental, 2016, 180, 674-679.
[56]J. Chang, L. Liang, C. Li, M. Wang, J. Ge, C. Liu and W. Xing, Green Chemistry, 2016, 18, 2287-2295.
[57]L. Zeng and T. S. Zhao, Nano Energy, 2015, 11, 110-118.
[58]J. Parrondo, M. George, C. Capuano, K. E. Ayers and V. Ramani, Journal of Materials Chemistry A, 2015, 3, 10819-10828.

[59]T. Pandiarajan, L. John Berchmans and S. Ravichandran, RSC Advances, 2015, 5, 34100-34108.
[60]C. C. Pavel, F. Cecconi, C. Emiliani, S. Santiccioli, A. Scaffidi, S. Catanorchi and M. Comotti, Angewandte Chemie International Edition, 2014, 53, 1378-1381.
[61]S. H. Ahn, B.-S. Lee, I. Choi, S. J. Yoo, H.-J. Kim, E. Cho, D. Henkensmeier, S. W. Nam, S.-K. Kim and J. H. Jang, Applied Catalysis B: Environmental, 2014, 154-155, 197-205.
[62]X. Wu and K. Scott, International Journal of Hydrogen Energy, 2013, 38, 3123-3129.
[63]Y.-C. Cao, X. Wu and K. Scott, International Journal of Hydrogen Energy, 2012, 37, 9524-9528.
[64]M. Faraj, M. Boccia, H. Miller, F. Martini, S. Borsacchi, M. Geppi and A. Pucci, International Journal of Hydrogen Energy, 2012, 37, 14992-15002.
[65]Y. Leng, G. Chen, A. J. Mendoza, T. B. Tighe, M. A. Hickner and C.-Y. Wang, Journal of the American Chemical Society, 2012, 134, 9054-9057.
[66]L. Xiao, S. Zhang, J. Pan, C. Yang, M. He, L. Zhuang and J. Lu, Energy & Environmental Science, 2012, 5, 7869-7871.
[67]X. Wu and K. Scott, Journal of Power Sources, 2012, 214, 124-129.
[68]X. Wu and K. Scott, Journal of Materials Chemistry, 2011, 21, 12344-12351.
